# From Testis to Retroperitoneum: The Role of Radiomics and Artificial Intelligence for Primary Tumors and Nodal Disease in Testicular Cancer: A Systematic Review

**DOI:** 10.3390/medicina62010125

**Published:** 2026-01-07

**Authors:** Teodora Telecan, Vlad Cristian Munteanu, Adriana Ioana Gaia-Oltean, Carmen-Bianca Crivii, Roxana-Denisa Capraș

**Affiliations:** 1 Department of Anatomy and Embryology, “Iuliu Hațieganu” University of Medicine and Pharmacy, 400012 Cluj-Napoca, Romania; t.telecan@gmail.com (T.T.); bianca.crivii@umfcluj.ro (C.-B.C.); capras.roxana@umfcluj.ro (R.-D.C.); 2 Department of Pathology, County Emergency Clinical Hospital, 400347 Cluj-Napoca, Romania; 3 Department of Urology, The Oncology Institute “Prof Dr. Ion Chiricuta”, 400015 Cluj-Napoca, Romania; 4 Department of Obstetrics and Gynecology, University of Medicine and Pharmacy Iuliu Hatieganu, 400347 Cluj-Napoca, Romania; oltean_adriana_ioana@yahoo.com; 5 Department of Obstetrics and Gynecology, “Regina Maria” Hospital, 400609 Cluj-Napoca, Romania

**Keywords:** testicular cancer, radiomics, texture analysis, artificial intelligence, retroperitoneal lymph nodes, chemotherapy response, diagnostic imaging

## Abstract

*Background and Objectives*: Radiomics and artificial intelligence (AI) offer emerging quantitative tools for enhancing the diagnostic evaluation of testicular cancer. Conventional imaging—ultrasound (US), magnetic resonance imaging (MRI), and computed tomography (CT)—remains central to management but has limited ability to characterize tumor subtypes, detect occult nodal disease, or differentiate necrosis, teratoma, and viable tumor in post-chemotherapy residual masses. This systematic review summarizes current advances in radiomics and AI for both primary tumors and retroperitoneal disease. *Materials and Methods*: A systematic search of PubMed, Scopus, and Web of Science identified studies applying radiomics or AI to testicular tumors, retroperitoneal lymph nodes and post-chemotherapy residual masses. Eligible studies included quantitative imaging analyses performed on ultrasound, MRI, and CT, with optional integration of clinical or molecular biomarkers. Eighteen studies met inclusion criteria and were evaluated with respect to methodological design, diagnostic performance, and translational readiness. *Results*: Across modalities, radiomics demonstrated encouraging discriminatory capacity, with accuracies of 74–82% for ultrasound, 80.7–97.9% for MRI, and 71.7–85.3% for CT. CT-based radiomics for post-chemotherapy residual masses showed moderate ability to distinguish necrosis/fibrosis, teratoma, and viable germ-cell tumor, though heterogeneous methodologies and limited external validation constrained generalizability. The strongest performance was observed in multimodal approaches: integrating radiomics with clinical variables or circulating microRNAs improved accuracy by up to 12% and 15%, respectively, mirroring gains reported in other oncologic radiomics applications. Persisting variability in segmentation practices, acquisition protocols, feature extraction, and machine-learning methods highlights ongoing barriers to reproducibility. *Conclusions*: Radiomics and AI-enhanced frameworks represent promising adjuncts for improving the noninvasive evaluation of testicular cancer, particularly when combined with clinical or molecular biomarkers. Future progress will depend on standardized imaging protocols, harmonized radiomics pipelines, and multicenter prospective validation. With continued methodological refinement and clinical integration, radiomics may support more precise risk stratification and reduce unnecessary interventions in testicular cancer.

## 1. Introduction

Testicular tumors represent the most frequent solid malignancy in male patients between 15 and 44 years of age, accounting for approximately 1–2% of all male cancers and an estimated 75,000 new cases being annually diagnosed worldwide [1]. The 2022 World Health Organization (WHO) classification [2] defines testicular tumors predominantly as germ cell tumors, which account for 90–95% of cases and include seminoma—the most frequent subtype, representing 52–56% [3]. The non-seminomatous germ cell tumor (NSGCT) category comprises embryonal carcinoma, postpubertal yolk sac tumor, choriocarcinoma, postpubertal teratoma, mixed germ cell tumors, and trophoblastic tumors. In contrast, non-germ cell testicular neoplasms form a separate and less common group that includes sex cord–stromal tumors, mixed germ–sex cord tumors, neuroendocrine tumors, and mesothelial tumors.

Initial imaging assessment of a suspected primary testicular tumor relies on scrotal ultrasonography (US), which remains the recommended first-line modality owing to its wide availability and high sensitivity. However, interpretation of sonographic features is inherently operator dependent, and although seminomas can be identified with accuracies approaching 90%, the characterization of non-seminomatous histologies is substantially less reliable [4]. In cases where ultrasonographic findings are indeterminate, scrotal magnetic resonance imaging (MRI) may be employed as an adjunct problem-solving examination, offering superior soft-tissue contrast and improving discrimination between benign and malignant lesions; nevertheless, its use is generally reserved for equivocal presentations [5]. For staging purposes, the American Urological Association recommends contrast-enhanced cross-sectional imaging of the abdomen and pelvis with computed tomography (CT), as the retroperitoneum represents the most frequent site of metastatic dissemination [6]. Contrast-enhanced CT demonstrates an accuracy of approximately 83–84% for detecting retroperitoneal lymph node involvement [1]; however, its utility in assessing post-chemotherapy residual masses in NSGCT is limited, as CT findings cannot reliably distinguish necrosis or fibrosis from teratoma or viable tumor in approximately 50–67% of residual masses larger than 1 cm [7].

Importantly, several key management decisions in testicular cancer hinge on accurate preoperative and post-treatment imaging assessment, including the extent of surgical intervention and the indication for retroperitoneal lymph node dissection. In this setting, imaging misclassification may result in unnecessary invasive procedures in patients with benign lesions, favorable histologies, or post-chemotherapy necrosis, while insufficient characterization of viable disease may delay appropriate treatment escalation. These diagnostic challenges emphasize the need for imaging approaches that move beyond lesion detection and support more refined risk stratification at clinically relevant decision points.

In the light of the current imagistic limitations, radiomics has emerged as a promising quantitative approach capable of extending the diagnostic value of conventional imaging. This domain refers to the high-throughput extraction and analysis of large numbers of quantitative features from medical images, transforming routine scans into mineable, high-dimensional data that capture lesion heterogeneity, shape, and texture—often beyond what is discernible by human visual assessment [8]. Although introduced as a formal concept in 2012 [9], radiomics has only recently been applied to testicular cancer, with the first investigation published in 2018 by Lewin et al. [10]. Since then, all major imaging modalities—ultrasound, MRI, and contrast-enhanced CT—have been explored from a radiomic perspective, targeting both primary testicular tumors and retroperitoneal lymph nodes or post-chemotherapy residual masses. As a noninvasive preoperative tool, radiomics holds the potential to serve as a noninvasive surrogate to histopathology, offering insights into tumor biology that may assist clinicians in anticipating treatment requirements and refining therapeutic strategies.

The objective of this systematic review is to critically synthesize current evidence on radiomics and artificial intelligence applications in testicular cancer imaging. Specifically, it aims to evaluate radiomics-based approaches applied to the assessment of primary testicular tumors and to the characterization of retroperitoneal lymph nodes and post-chemotherapy residual masses, as well as emerging multimodal models integrating imaging-derived features with clinical or molecular biomarkers. By summarizing methodological design, diagnostic targets, and validation strategies, this review seeks to clarify the current state of the field and identify key challenges to clinical translation.

## 2. Materials and Methods

The present systematic review was conducted in accordance with the PRISMA 2020 guidelines. The review protocol was not prospectively registered, as this review was designed as an exploratory synthesis of emerging radiomics applications rather than a hypothesis-driven meta-analysis. A comprehensive literature search was performed independently by two reviewers (T.T. and V.C.M.) in PubMed, Scopus, and Web of Science (WoS) to identify studies evaluating the diagnostic accuracy of texture analysis features and artificial intelligence pipelines in testicular cancer. Titles and abstracts were screened independently by the same two reviewers, followed by full-text eligibility assessment. Any discrepancies regarding study inclusion were resolved through discussion and consensus, with arbitration by a third, senior reviewer (C.-B.C.) when necessary. Blinding of reviewers to study authors, institutions, or journal sources was not applied during screening, data extraction, or risk of bias assessment, as such information is intrinsic to published studies; however, predefined extraction forms and standardized assessment criteria were used to minimize subjective bias. Searches in PubMed, Scopus, and Web of Science were conducted in parallel and completed within the same search period, with all databases last queried on 7 September 2025. Only studies with full text available in English were considered eligible for inclusion.

The PubMed strategy combined both MeSH terms and free-text keywords related to testicular neoplasms and radiomics/AI techniques: (“Testicular Neoplasms”[Mesh] OR “testicular cancer” OR “testicular tumor*” OR “germ cell tumor*” OR seminoma OR nonseminoma) AND (“Radiomics”[Mesh] OR radiomic* OR “texture analysis” OR “machine learning” OR “deep learning” OR “artificial intelligence” OR “computer-assisted diagnosis” OR CAD). The Scopus query was constructed as follows: TITLE-ABS-KEY(“testicular cancer” OR “testicular tumor*” OR “testicular neoplasm*” OR “germ cell tumor*” OR seminoma OR nonseminoma) AND TITLE-ABS-KEY(radiomic* OR “texture analysis” OR “machine learning” OR “deep learning” OR “artificial intelligence” OR “computer assisted diagnosis” OR CAD). The Web of Science search used a Topic (TS) query: TS=(“testicular cancer” OR “testicular tumor*” OR “testicular neoplasm*” OR “germ cell tumor*” OR seminoma OR nonseminoma) AND TS=(radiomic* OR “texture analysis” OR “machine learning” OR “deep learning” OR “artificial intelligence” OR “computer assisted diagnosis” OR CAD). No date limits were applied at the database search stage; the restriction to studies published within the past 10 years (2015–2025) was implemented manually during title/abstract and full-text screening, in accordance with the PRISMA flow diagram, to ensure relevance to contemporary imaging technology and clinical practice. The 10-year time window was selected to capture studies reflecting contemporary imaging technology and modern radiomics methodology. Although texture analysis has been explored earlier in medical imaging, radiomics was formally introduced as a distinct, high-throughput analytical framework in 2012, and its widespread application—particularly incorporating standardized feature extraction, machine-learning pipelines, and reproducibility considerations—has largely emerged within the past decade. Earlier texture-analysis studies, which predate current radiomics workflows and IBSI-compliant feature definitions, were therefore considered outside the scope of this review. This temporal restriction was applied consistently across all imaging modalities and diagnostic tasks. To enhance the comprehensiveness of the search, the reference lists of all included articles were manually screened. Any discrepancies between reviewers were resolved by discussion, with unresolved cases adjudicated by the senior author.

Studies were eligible if they evaluated the performance of radiomic parameters or artificial intelligence-based decision-support tools in characterizing primary testicular masses, as well as studies assessing chemotherapy response by differentiating retroperitoneal lymph node fibrosis from metastatic spread. In both instances, the histopathological report from orchiectomy or lymphadenectomy was used as the reference standard. Inclusion was restricted to original studies published in peer-reviewed journals, with full text accessible in English.

The following criteria were applied for study exclusion:

- Full-text available in languages other than English;
- Designed as a systematic review, meta-analysis, comment, letter to the editor, or meeting abstract;
- Published before 2015;
- Conducted on animal or experimental models.

Data extraction was performed independently by two reviewers using a predefined, standardized data extraction form. Extracted variables included study characteristics (year of publication, study design, sample size, and population), imaging modality and acquisition parameters, segmentation approach, radiomics workflow (feature extraction, feature selection, and machine-learning or deep-learning model type), integration of clinical or molecular variables, reference standard, validation strategy, and reported performance metrics (including accuracy, sensitivity, specificity, and AUC when available). Where reported, information on feature standardization, model calibration, or decision-curve analysis was also recorded. Any discrepancies in extracted data were resolved through discussion and consensus by the 2 reviewers, with arbitration by the forementioned senior reviewer when necessary. Given the substantial heterogeneity across studies, results were synthesized qualitatively.

During eligibility assessment, we excluded studies that focused primarily on the surgical management of testicular tumors or that investigated prognostic markers other than imaging-based features, such as circulating microRNAs or histological, tumor microenvironment factors. We also excluded papers centered on other malignancies than testicular tumors, as well as those lacking a clearly described radiomic protocol.

**Figure 1:**
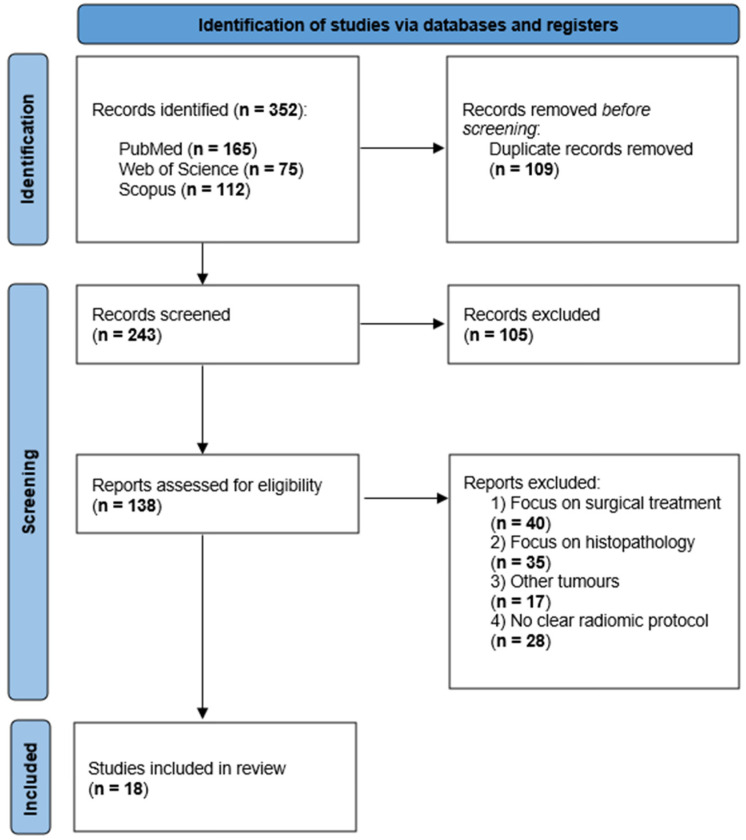
PRISMA flow diagram of the study selection process. The database search identified 352 records, of which 243 remained after removal of duplicates. Following title and abstract screening, 105 records were excluded. Full-text eligibility assessment was conducted for 138 articles, resulting in the exclusion of 120 studies based on predefined criteria, including a primary focus on surgical management, histopathological or biochemical analyses without imaging-based radiomics, non-testicular malignancies, or the absence of a clearly described radiomics protocol. Ultimately, 18 studies were included in the qualitative synthesis.

The search process was synthetized in a Preferred Reporting Items for Systematic Reviews and Meta-Analyses (PRISMA) type flowchart (Figure 1), and a completed PRISMA checklist is provided in the Supplementary File S1.

The methodological quality and risk of bias of the included studies were appraised using the Quality Assessment of Diagnostic Accuracy Studies-2 (QUADAS-2) framework [11]. QUADAS-2 is a validated tool specifically designed to assess the methodological quality and risk of bias in diagnostic accuracy studies. It evaluates four key domains—patient selection, index test, reference standard, and flow and timing—each assessed for risk of bias, with the first three domains additionally evaluated for concerns regarding applicability. In the present review, QUADAS-2 was applied independently by two reviewers to all included studies based on predefined signaling questions, with disagreements resolved through discussion and, when necessary, arbitration by the senior author. The assessment was used to provide a structured overview of methodological limitations across studies rather than to exclude studies based on predefined quality thresholds.

No formal sensitivity or subgroup analyses were performed. Given the substantial heterogeneity across studies in terms of imaging modalities, radiomics pipelines, machine learning models, and outcome definitions, quantitative sensitivity analyses or subgroup comparisons were not considered methodologically appropriate for this qualitative synthesis.

## 3. Results

### 3.1. Study Selection and Characteristics

The initial search yielded 352 records, of which 243 remained after removal of duplicates. Title and abstract screening led to the exclusion of 105 records due to lack of relevance (e.g., animal studies), language restrictions, non-originality, or publication date, leaving 138 articles for full-text eligibility assessment.

Full-text screening resulted in the exclusion of 120 studies: 40 concerned orchiectomy or retroperitoneal lymphadenectomy from a surgical point of view exclusively, 35 evaluated biochemical or histopathological markers, 17 addressed malignancies of non-testicular origin, and 28 did not include a clearly defined radiomics methodology.

In total, 18 studies met the inclusion criteria and were included in the qualitative synthesis. Of these, 3 investigated ultrasound-based approaches and 7 applied radiomics to magnetic resonance imaging of primary testicular tumors, while 8 evaluated computed tomography with a focus on retroperitoneal lymph node. Out of these, six studies integrated textural features with additional variables, such as circulating microRNAs and/or clinical parameters, to build combined predictive models.

**Figure 2:**
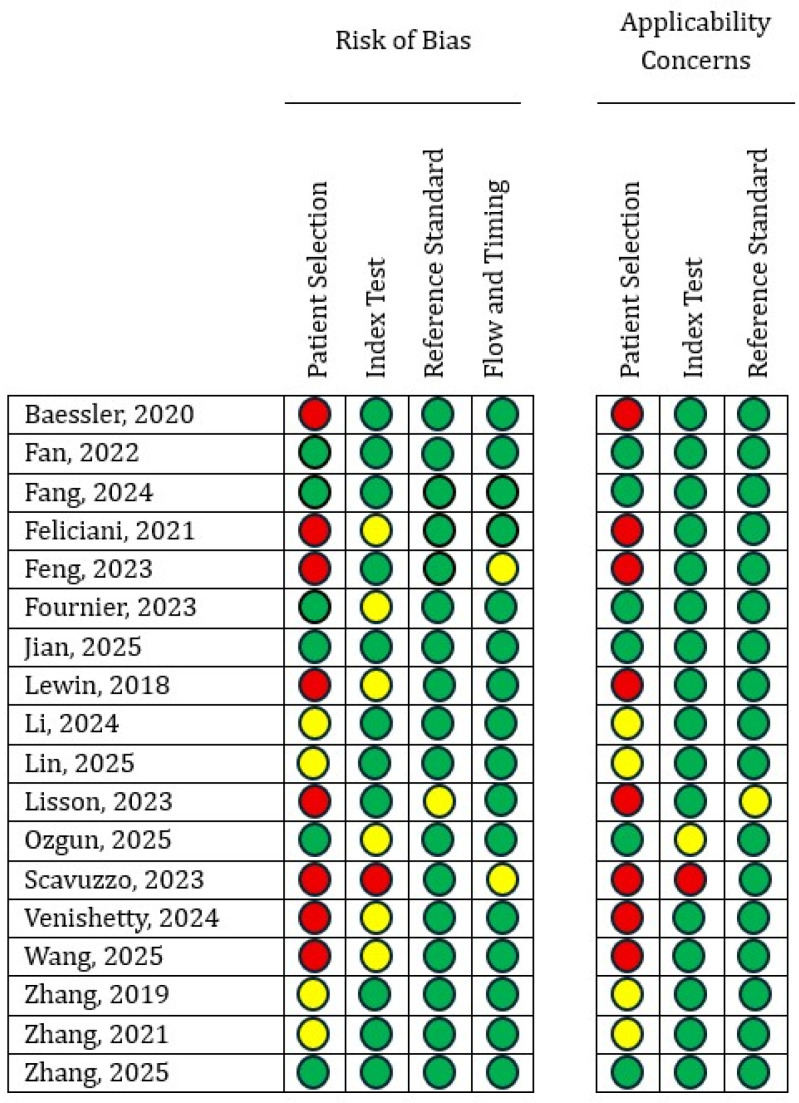
Detailed QUADAS-2 evaluation of methodological quality for each study assessing the diagnostic accuracy of the included radiomics models [10,12,13,14,15,16,17,18,19,20,21,22,23,24,25,26,27,28]. Green = Low bias risk; Yellow = Unclear bias risk; Red = High bias risk.

**Figure 3:**
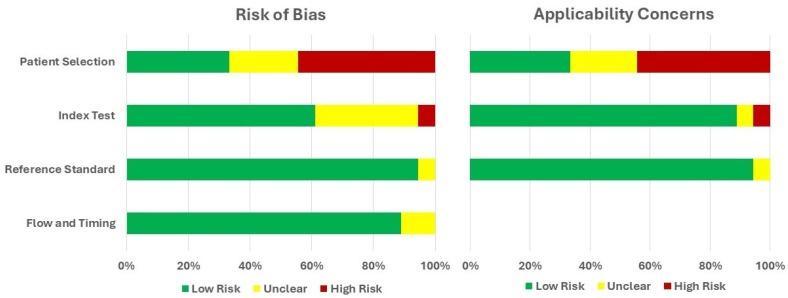
Summary of QUADAS-2 assessments across all included studies.

The QUADAS-2 risk of bias assessment is summarized in Figure 2 (per-study evaluation) and Figure 3 (domain-level overview). Across the included studies, high or unclear risk of bias most commonly arose in the patient selection and index test domains. Most studies showed a low risk of bias in the reference standard and flow-and-timing domains. However, concerns were more frequently identified in the patient selection and index test domains, largely reflecting retrospective study designs, restrictive or incompletely described inclusion criteria, variability in radiomics workflows, and insufficient reporting of blinding procedures. Applicability concerns mirrored this pattern, with the highest issues observed once more in patient selection, while index test and reference standard applicability remained largely acceptable across studies.

**Figure 4:**
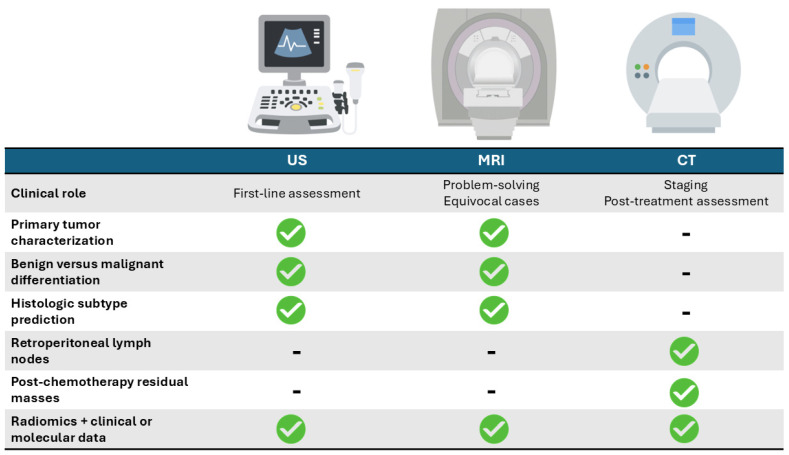
Conceptual overview of radiomics and artificial intelligence applications in testicular cancer imaging across ultrasound (US), magnetic resonance imaging (MRI), and computed tomography (CT). Checkmarks indicate clinical contexts addressed by radiomics-based approaches in the included studies, while dashes indicate applications not evaluated. The first row summarizes the typical clinical role of each imaging modality within the diagnostic pathway.

To facilitate navigation of the subsequent modality-specific analyses, a conceptual overview of radiomics applications across ultrasound, magnetic resonance imaging, and computed tomography is provided in Figure 4.

### 3.2. Primary Tumor Assessment

#### 3.2.1. Ultrasound-Based Radiomics

A total of 3 studies met the inclusion criteria and investigated the diagnostic performance of ultrasound-based radiomics for the characterization of primary testicular tumors. The main characteristics and findings of these studies are summarized in Table 1. All included studies were retrospective in design. Two were multicenter investigations and implemented both internal and external validation strategies. Sample sizes ranged from 148 to 489 cases, with 464 to 1561 features extracted from each region of interest. All papers reported manual segmentation of the regions of interest. Histopathology from orchiectomy served as the reference standard in all studies, while one incorporated histopathology reports from both diagnostic biopsies and radical procedures.

Among the ultrasound-based radiomics studies included in this review, Fang et al. [12] developed an ultrasound-based deep learning radiomics (DLR) pipeline that combined standard radiomic descriptors with features extracted from a fine-tuned ResNet-50 network, using a Minimum Redundancy Maximum Relevance and Least Absolute Shrinkage and Selection Operator (mRMR–LASSO) combined selection strategy to create compact predictive signatures for both diagnostic tasks. For the classification of tumoral versus adjacent tissue, the final model incorporated 7 radiomic and 19 deep-learning features and achieved excellent discriminative performance, with AUCs of 0.954 in the training set, 0.850 in the internal validation cohort, and 0.803 in the external test cohort. In the benign versus malignant task, the optimized DLR model relied on 4 radiomic and 20 deep-learning features and maintained similarly strong accuracy, yielding AUCs of 0.894, 0.823, and 0.799 across the same three cohorts. Importantly, both models demonstrated stable generalization during external validation despite differences in ultrasound equipment and acquisition settings between centers, underscoring the robustness of the hybrid radiomics–deep learning approach.

As a second level of analysis, the included studies extended ultrasound-based radiomics beyond the benign–malignant distinction to explore whether quantitative image features can further characterize malignant tumor subtypes. In the germ cell versus non-germ cell tumor setting, Lin et al. [13] valuated whether grayscale texture patterns captured from conventional ultrasound could distinguish classic germ-cell neoplasms from a diverse group of non-germ cell tumors, including Leydig, Sertoli, adenomatoid, hemangioma and other sex cord–stromal lesions. Using manually segmented grayscale ultrasound images, the authors extracted 464 radiomics features, retained 419 after Intraclass Correlation Coefficient (ICC) filtering, and reduced dimensionality via an mRMR–LASSO pipeline to build an 8-feature radiomics signature. The resulting germ cell versus non-germ cell model achieved accuracies of 81% in the training set and 82% in the validation cohort.

Building on this, both Lin et al. [13] and Zhang et al. [14] further investigated whether ultrasound radiomics could discriminate seminomatous from non-seminomatous germ cell tumors. In Lin et al.’s single-center study [13], radiomics analysis of conventional grayscale ultrasound provided only modest subtype separability, with performance stabilizing around an AUC of 0.74 in both training and validation, reflecting the substantial overlap in ultrasound appearance between these entities and the constraints of native-resolution imaging. In contrast, Zhang et al. [14] employed a multicenter design and incorporated a deep learning-based super-resolution (SR) reconstruction step prior to radiomics feature extraction. Using a Generative Adversarial Network (GAN)-based model to upscale the native ultrasound images, the SR pipeline enhanced spatial detail by restoring fine textural and structural information that is typically lost in conventional ultrasound reconstruction, thereby providing a richer basis for radiomic analysis. This image-enhancement step markedly improved the separability of seminomatous and non-seminomatous tumors, with the resulting SR radiomics classifier achieving AUCs of 0.90, 0.82, and 0.91 in the training, domestic validation, and international validation cohorts, respectively. Notably, the SR model not only outperformed the corresponding native-resolution radiomics models but also exceeded the diagnostic performance of two experienced radiologists, whose AUCs ranged from 0.77 to 0.85 across validation cohorts. These findings highlight that deep learning-based image enhancement can reveal discriminative textural signals imperceptible to human observers, enabling radiomics models to surpass expert visual interpretation in subtype classification.

#### 3.2.2. MRI-Based Radiomics

A total of seven studies met the inclusion criteria and evaluated MRI-based radiomics for the characterization of primary testicular tumors. The main characteristics and findings of these studies are summarized in Table 2. All investigations were retrospective and single-center in design. Sample sizes ranged from 39 to 148 cases, with 66 to 1781 radiomic features extracted per region of interest. All studies relied on manual segmentation. Most papers (85.71%, *n* = 6) extracted textural features from a single MRI sequence, and among these, the majority (83.33%, *n* = 5) used T2-weighted images as the primary source for radiomic analysis. Scanning protocols were based on 3 Tesla MRI systems in 71.42% (*n* = 5) of the included papers. Histopathology from orchiectomy served as the sole reference standard in 42.85% (*n* = 3) of the studies, whereas the remaining 57.14% (*n* = 4) incorporated results from both diagnostic testicular biopsies and radical surgical specimens. Regarding diagnostic targets, 83.33% of the studies (*n* = 5) investigated radiomics models for distinguishing benign from malignant lesions, while a smaller subset (28.57%, *n* = 2) focused on differentiating specific malignant tumor subtypes.

Across the seven MRI-based radiomics studies included in this review, five investigations specifically evaluated whether quantitative MRI texture analysis can differentiate benign from malignant testicular lesions. The benign comparator groups were diverse and clinically representative, commonly including entities such as epidermoid cysts, testicular infarction, orchitis, abscesses, hematomas, Leydig and Sertoli cell tumors, adenomatoid tumors, dermoid cysts, hemangiomas, and sex cord–stromal tumors, depending on the study. Reported diagnostic performance varied substantially across papers but generally demonstrated moderate-to-high accuracy, with test accuracies ranging between 78.4% and 90.5%, reflecting the capacity of MRI-based texture patterns to capture relevant differences in lesion heterogeneity.

In terms of the employed MRI acquisition, a subgroup of studies focused exclusively on T2-weighted radiomics yet implemented markedly different analytical strategies. Zhang et al. [17] evaluated individual histogram and Intra-Peri-lesional Radiomic Spatial transition (Ipris) texture features using univariate statistical testing and ROC analysis rather than multivariate machine-learning models, identifying several histogram descriptors as the strongest discriminators. The study conducted by Feng et al. [19], in contrast, systematically compared ten supervised machine-learning classifiers and demonstrated that boosting-based algorithms—particularly XGBoost—achieved superior diagnostic performance, 98.7% and 90.5% in the training and validation phases, respectively. Wang et al. [21] further broadened the methodological landscape by adopting the Tree-based Pipeline Optimization Tool, which automatically builds and improves machine-learning pipelines using a step-by-step trial-and-refinement procedure, eliminating the need for manual selection of algorithms and parameters. Although this automated approach produced a highly optimized model with a training accuracy of 94.2%, performance declined substantially on the independent validation set (78.4%), indicating that the model may have learned patterns specific to the training group rather than general tumor characteristics, and reinforcing the necessity of external validation to confirm that the results are broadly applicable.

In addition to T2-weighted radiomics, one study evaluated whether diffusion-based texture analysis could improve lesion characterization using apparent diffusion coefficient maps alone. Fan et al. [18] extracted whole-lesion ADC radiomic features and built a compact six-feature signature that achieved good discrimination between benign and malignant lesions, with a validation accuracy of 86.8%, comparable to the performance observed in the T2-based approaches. Notably, ADC radiomics outperformed conventional diffusion metrics such as mean and minimum ADC, indicating that microstructural heterogeneity captured through texture analysis may provide added diagnostic value beyond standard diffusion measurements. Beyond single-sequence analyses, Jian et al. [20] provided the only true multiparametric MRI radiomics approach, integrating features extracted from T2-weighted imaging, diffusion-weighted imaging, ADC maps, and contrast-enhanced T1-weighted acquisitions. After constructing radiomics signatures from each sequence individually and from multiple combined configurations, the full four-sequence model demonstrated the strongest performance, achieving a validation accuracy of 81.0% and an AUC of 0.885. These results indicate that, while combining structural, diffusion, and contrast-enhanced sequences may provide a richer representation of lesion heterogeneity, the advantage over the strongest T2-weighted models was relatively small, potentially because the dataset was not large enough to support the much higher feature complexity introduced by the multiparametric approach.

An additional focus of the MRI-based radiomics literature was the separation of germ cell from non-germ cell tumors. Feliciani et al. [16] conducted the first MRI-based radiomics study specifically aimed at distinguishing germ cell from non-germ cell tumors using T2-weighted imaging alone. After manual segmentation, approximately 500 radiomic features were extracted and reduced through LASSO selection to a compact three-feature signature. This signature was then used to train a linear Support Vector Machine classifier, evaluated using fivefold cross-validation. The resulting model achieved an overall accuracy of 89% for differentiating TGCT from TNGCT, correctly separating germ cell tumors from a diverse range of non-germ cell histologies, including Leydig, Sertoli, adenomatoid, and epidermoid tumors.

A further line of investigation examined whether MRI-based radiomics could support the preoperative distinction between seminomatous and non-seminomatous germ cell tumors. Both Feliciani et al. [16] and Zhang et al. [15] focused on T2-weighted imaging and developed concise radiomic signatures intended to capture microstructural heterogeneity within the tumor, which typically remains undetectable through conventional MRI assessment. Feliciani et al. [16] employed a stability-filtered feature set combined with a linear support vector machine, achieving an accuracy of 86%, whereas Zhang et al. [15] applied an mRMR–LASSO feature selection pipeline followed by logistic regression, yielding an AUC of 97.9% with a sensitivity of 90% and a specificity of 100%. Although the reported performance metrics appear promising, these findings should be interpreted with caution, as both studies were conducted on very small cohorts, representing the smallest datasets within the MRI radiomics subgroup.

### 3.3. CT-Derived Radiomics for Retroperitoneal Nodal Metastasis Detection and Post-Chemotherapy Residual Mass Assessment

Eight studies met the inclusion criteria and evaluated CT-based radiomics for the characterization of retroperitoneal lymph nodes and post-treatment residual masses in patients with testicular cancer. The main characteristics and findings of these studies are summarized in Table 3. Across all studies, 87.5% (*n* = 7) were fully retrospective, while one study (12.5%) used a retrospective design for model development with a prospective cohort for external validation. Regarding study setting, 75% (*n* = 6) were conducted at a single center, whereas the remaining 25% (*n* = 2) implemented multicenter designs. Sample sizes ranged from 45 to 273 cases and reported radiomic feature counts varied between 85 and 1130 per region of interest. Seventy-five percent (*n* = 6) of studies relied on manual segmentation performed by radiologists, whereas the remaining 25% (*n* = 2) used a semi-automated approach in which initial lesion contours were generated in 3D Slicer and subsequently refined through manual correction. Histopathology from retroperitoneal lymph node or residual mass dissection served as the reference standard in 75% (*n* = 6) of the studies, while one investigation used the resection report of the retroperitoneal residual mass as its histological endpoint. The remaining study relied on the orchiectomy specimen for initial tumor confirmation and determined relapse solely through consecutive CT scans during a structured 5-year imaging follow-up period, without surgical verification of the retroperitoneal findings. Seventy-five percent of studies (*n* = 6) aimed to differentiate among teratoma, viable germ cell tumor, and fibrosis or necrosis, whereas the remaining 25% (*n* = 2) focused specifically on distinguishing seminomatous from non-seminomatous germ cell tumors and teratoma versus non-teratoma retroperitoneal residual masses.

Across the six CT-based radiomics studies included in this review that specifically aimed to distinguish post-chemotherapy fibrosis/necrosis from residual teratoma and viable germ-cell tumor, substantial methodological heterogeneity was observed. Although all studies used contrast-enhanced CT as the imaging backbone, analytic approaches ranged from simple first-order texture comparisons to multi-feature, model-based radiomics classifiers. As such, diagnostic performance was generally moderate, with reported validation accuracies ranging between 71.7% and 85.3%.

Among these papers, the study conducted by Venishetty et al. [27] stands out as the only radiomics investigation that produced largely negative results, with quantitative CT features failing to meaningfully discriminate between benign and malignant residual masses. In this analysis, only the largest lymph node was segmented per patient, and feature comparisons were limited to first-order and shape descriptors, without the development of a multi-feature radiomics signature or machine-learning classifier. Under this simplified framework, only four highly collinear first-order intensity metrics reached statistical significance, while all texture- and shape-based features were non-informative. These findings suggest that restricted sampling and minimal analytic pipelines may markedly limit the ability of CT radiomics to capture relevant histopathologic differences.

Extending the methodological scope, Lewin et al. [10] systematically evaluated whether restricting lesions by size improved the discriminatory performance of their radiomics classifier. Accuracy varied across axial-diameter thresholds, ranging from 62.4% at <40 mm to 69.4% at <20 mm, but the best overall performance was consistently achieved when no size restriction was applied (71.7% accuracy, AUC 0.74). Similar patterns were observed when stratifying by effective radius, with AUCs increasing from 0.57 (radius < 15 mm) to 0.70 (<25 mm), yet never exceeding the unrestricted dataset. These results indicate that although narrower size subsets produced modest fluctuations in accuracy, size-based filtering did not enhance model discriminability beyond that achieved using the full cohort.

On the opposite end of the performance spectrum, two studies demonstrated notably rigorous validation strategies, each incorporating independent external cohorts. Baessler et al. [22] analyzed 204 lymph nodes from 80 patients using per-node histopathologic matching, which provided high granularity in ground-truth labeling. Imaging data were acquired across four CT vendors, introducing substantial heterogeneity in acquisition parameters; nevertheless, the model maintained stable performance, with an external validation accuracy of 81% (sensitivity of 84% and specificity of 78%). Similarly, Li et al. [26] developed a multistep CT-based radiomics pipeline trained on a retrospective cohort and subsequently evaluated in a prospective external validation set, achieving a macro-averaged AUC of 0.91 (95% CI 0.80–0.99) and an overall accuracy of 81%. This study, which included 139 patients and 187 residual masses, remains the only investigation to date to prospectively validate a radiomics classifier for post-chemotherapy residual masses. To support clinical translation, the authors also implemented a publicly accessible, web-based dynamic nomogram integrating radiomic scores with clinical variables to generate individualized probability estimates for necrosis/fibrosis, teratoma, or viable germ-cell tumor.

Beyond the characterization of post-chemotherapy residual masses, two studies extended radiomics analysis toward differentiating specific tumor subtypes. Ozgun et al. [28] developed a CT-based radiomics model aimed specifically at distinguishing teratoma from non-teratoma residual masses in metastatic non-seminomatous germ-cell tumors. Using manually segmented lesions ≥ 10 mm (111 lesions from 52 patients), the authors applied LASSO-based feature selection followed by CatBoost classification. In the radiomics-only configuration, the model achieved an accuracy of 89% in the training set and 81% in the independent test set, with corresponding test-set sensitivity and specificity of 76% and 65%, respectively, thus indicating moderate discriminative capacity for identifying residual retroperitoneal teratoma lesions. In a separate line of investigation, Lisson et al. [24] examined radiomics in a markedly different clinical context—early-stage testicular cancer—rather than in post-chemotherapy disease. Across 273 manually segmented retroperitoneal lymph nodes from 91 patients, 85 IBSI-compliant radiomic features were extracted from baseline contrast-enhanced CT to evaluate nodal texture patterns associated with seminomatous versus non-seminomatous disease biology. Unlike studies relying on RPLND or metastasectomy for histologic ground truth, the authors used orchiectomy histology combined with five-year longitudinal CT follow-up to determine metastatic status. Under this framework, radiomics-only models reached an accuracy of 83% on held-out testing, suggesting that nodal texture descriptors convey subtype-related differences even in the absence of surgical confirmation. Together, these investigations demonstrate that radiomics may assist in subtype-focused risk stratification across distinct clinical settings, from residual mass evaluation to early-stage surveillance, and could potentially reduce unnecessary RPLND procedures by approximately 41% [23].

Taken together, the modality-specific analyses highlight heterogeneous diagnostic tasks and performance metrics across ultrasound-, MRI-, and CT-based radiomics studies. To synthesize these findings, Table 4 provides a compact summary of the principal diagnostic tasks and best-reported performance metrics across imaging modalities, prior to discussing combined and multimodal approaches.

### 3.4. Integrated Models Combining Radiomics with Clinical and Molecular Data

Across the studies included in this review, six investigations explored combined predictive approaches, integrating radiomics with additional clinical or molecular information. Of these, two were based on ultrasound, one on MRI, and three on CT imaging. Four studies incorporated clinical variables—most consistently age, AFP, β-hCG, and BMI—reflecting established prognostic markers in testicular cancer. Two studies further extended this multimodal framework by integrating circulating microRNAs, specifically miR-371a-3p and miR-375-5p, thereby adding a molecular dimension to radiomics-based prediction. Table 5 summarizes the additional characteristics and combined-model findings from the studies reviewed above.

Across the four studies that evaluated clinically enhanced radiomics models, the integration of clinical variables consistently improved predictive performance compared with radiomics alone, irrespective of the classifier architecture employed. Lisson et al. [24] incorporated age, alpha-fetoprotein (AFP), beta-human chorionic gonadotropin (β-hCG), body mass index (BMI), and histology into a Random Forest model, increasing accuracy from 83% to 87% for predicting occult metastases in early-stage disease. Jian et al. [20] similarly reported gains using logistic regression, with the addition of tumor size to multiparametric MRI radiomics improving validation accuracy from 81% to 88.4%. In ultrasound-based workflows, Lin et al. [13] employed logistic regression and observed accuracy increases of 7% and 12% for TGCT versus TNGCT and SGCT versus NSGCT classification, respectively, after integrating clinical markers such as AFP, β-hCG, and tumor size. Fang et al. [12] used the most complex multimodal architecture, combining handcrafted radiomics, ResNet-50 deep-learning features, and clinical parameters within a deep learning-based fusion model, yielding a 10.6% improvement in external-validation accuracy for benign versus malignant discrimination. Collectively, these studies demonstrate that multimodal integration provides consistent incremental gains—typically between 4% and 12%—by leveraging complementary clinical and biological information alongside imaging-derived features.

Two studies investigated multimodal models that combined circulating microRNAs with CT-based radiomics, both employing machine-learning frameworks to integrate molecular and imaging features. Ozgun et al. [28] used a CatBoost classifier to merge radiomic descriptors with serum miR-371a-3p—a marker of viable germ-cell tumor (GCT)—and miR-375-5p, which has been associated with teratoma differentiation. This multimodal integration produced a substantial improvement in test-set performance, increasing accuracy from 81% to 96%. Li et al. [26] similarly incorporated circulating miR-371a-3p into a multistep radiomics pipeline based on logistic regression, achieving a marked enhancement in a prospectively collected external validation cohort, where the combined model reached an accuracy of 91%. Although the magnitude of improvement differed between the two investigations, both demonstrated that machine-learning integration of radiomics with biologically informative microRNAs provides complementary discriminatory value—between 10% and 15%—reflecting the distinct molecular pathways underpinning viable GCT and teratoma.

From a practical clinical perspective, the added value of multimodal integration lies not only in improved statistical performance, but in more actionable decision support. While radiomics alone captures imaging-based heterogeneity, the incorporation of serum tumor markers or circulating microRNAs anchors model predictions to biologically meaningful disease states. For example, integration of AFP and β-hCG may assist clinicians in distinguishing indolent from biologically active disease when imaging findings are equivocal, thereby refining risk stratification at initial diagnosis. Similarly, combining CT radiomics with miR-371a-3p or miR-375-5p has direct implications in the post-chemotherapy setting, where improved discrimination between viable tumor, teratoma, and necrosis could inform the decision to proceed with retroperitoneal lymph node dissection versus surveillance. In this context, multimodal models function as integrative decision-support tools that align imaging phenotypes with underlying tumor biology, offering more clinically interpretable and reliable guidance than radiomics-only approaches.

## 4. Discussion

Testicular cancer remains a clinically significant public health concern despite its relatively low incidence, primarily because it is the most common solid malignancy in a population otherwise at minimal risk for cancer, creating long-term implications for survivorship, fertility, psychosocial well-being, and societal productivity [3]. Although cure rates exceed 95% in early-stage disease [29], survivors face substantial treatment-related morbidity, including secondary malignant neoplasms—which occur at rates 2.1–2.6 times higher than in the general population following cisplatin-based chemotherapy or radiotherapy—as well as cardiovascular and metabolic complications, with metabolic syndrome nearly doubled in prevalence and hypertension or dyslipidemia affecting 40–55% of men beyond five years post-treatment [30]. Hypogonadism is reported in up to 45% of survivors, and fertility is significantly impacted, with a 10-year paternity rate approximately 30% lower than in age-matched controls, particularly among those treated for metastatic disease [31]. Overall, long-term toxicity is common: 37.6% of survivors experience at least three chronic health complications and 12.5% experience five or more within a median of 4.3 years after chemotherapy [3]. These enduring health burdens underscore the need for improved diagnostic precision and risk stratification to minimize overtreatment and its sequelae in this predominantly young patient population.

Despite their central role in the diagnostic pathway, conventional imaging techniques have important limitations in the evaluation of testicular cancer. Scrotal ultrasonography, although the first-line modality, demonstrates variable diagnostic performance, with accuracy ranging from 72% among less experienced operators to 83.5% in expert radiologists [32]. Multiparametric MRI offers improved soft-tissue characterization and higher specificity, particularly when ultrasound findings are indeterminate or when detailed local staging is needed for surgical planning, especially when testis-sparing surgery is considered. Reported sensitivity and specificity for distinguishing benign from malignant lesions reach 94.3% and 76.9%, respectively [33].

However, the most significant limitation of conventional imaging lies in the assessment of post-chemotherapy retroperitoneal residual masses. Computed tomography cannot reliably differentiate necrosis or fibrosis from teratoma or viable germ-cell tumor, leading to substantial overtreatment. Large series and meta-analyses consistently demonstrate that approximately 70% of patients undergoing retroperitoneal lymph node dissection after chemotherapy harbor only necrotic or fibrotic tissue, while 25% have teratoma and only 5% have viable malignancy. Consequently, up to 70% of post-chemotherapy RPLNDs may be histologically unnecessary, underscoring the need for more accurate, noninvasive imaging biomarkers capable of refining patient selection for surgery [34].

In this context, radiomics provides a quantitative framework capable of overcoming several limitations of conventional imaging by extracting reproducible, high-dimensional descriptors of lesion heterogeneity and morphology that extend beyond human visual assessment. Although its application to testicular cancer is as recent as 2018 [10], radiomics studies across ultrasound, MRI, and CT have consistently demonstrated encouraging discriminatory performance, with reported accuracies ranging from 74–82% for ultrasound, 80.7–97.9% for MRI, and 71.7–85.3% for CT.

In the post-chemotherapy setting, the diagnostic landscape remains especially challenging, and a comparison with established functional imaging further underscores this unmet need. Positron emission tomography-computed tomography (PET-CT) is the recommended modality only for seminoma residual masses, and its utility is confined to lesions ≥ 3 cm, where it achieves a high sensitivity (up to 95%) and a negative predictive value exceeding 90% for excluding viable tumor. However, its positive predictive value remains low (approximately 23–69%), limiting its ability to confirm malignancy in PET-avid lesions. Importantly, PET-CT is not recommended for initial staging or for non-seminomatous germ-cell tumors, as it cannot distinguish viable tumor or teratoma from necrosis or fibrosis [35]. These modality-specific constraints, together with size-dependent performance, highlight the persistent difficulty in accurately characterizing post-chemotherapy residual masses and illustrate why more advanced, quantitative imaging approaches—such as radiomics—are being explored to address these limitations.

A key insight emerging from recent work is that radiomics, while capturing complex patterns of lesion heterogeneity, does not fully encompass the multidimensional biological landscape of testicular cancer. Radiomic features quantify morphology and texture, but they remain agnostic to molecular activity, tumor marker secretion, and systemic cues that reflect tumor burden or differentiation. Clinical variables such as AFP, β-hCG, and tumor size, as well as circulating microRNAs, encode biological signals that are orthogonal to image-derived texture, providing complementary information that radiomics alone cannot infer.

Integrating these domains therefore produces a more complete representation of tumor phenotype, one that links macroscopic imaging heterogeneity to underlying biochemical and cellular processes. From a technical perspective, this multimodal architecture also enhances model stability: clinical and molecular features act as biologically constrained anchors that reduce the variance and overfitting tendencies of purely image-based classifiers, while improving calibration and generalizability across datasets.

Although radiomics is well positioned to complement traditional imaging by providing quantitative descriptors of tumor morphology and heterogeneity, its meaningful integration into clinical practice requires rigorous translational steps. To move beyond proof-of-concept studies, radiomics models must demonstrate reproducibility and stability across institutions, scanners, and patient populations, ideally through multicenter prospective validation cohorts that reflect real-world clinical variability.

Achieving this level of robustness remains challenging, given the considerable methodological heterogeneity observed across published studies. Variations in segmentation protocols, image acquisition parameters, reconstruction kernels, feature extraction settings, and machine-learning pipelines can all introduce non-biological variance that undermines reproducibility. Standardization efforts—including adherence to IBSI-compliant feature definitions [24] and statistical harmonization techniques—are therefore essential to reduce technical noise and ensure that extracted features reflect underlying tumor biology rather than scanner- or site-specific artifacts. Moreover, the absence of external validation in several studies raises concerns regarding overfitting and limits the generalizability of proposed models.

In this context, it is noteworthy that the multimodal approaches identified in our review—integrating radiomics with clinical variables or circulating microRNAs—yielded accuracy improvements of up to 12% and 15%, respectively. Although these models are recent and derive from relatively small cohorts, their performance gains closely parallel those reported in other malignancies, including lung, ovarian, and head-and-neck cancers, where combined radiomic-clinical or radiomic-molecular models typically achieve incremental improvements in the range of 7–15% [36,37,38]. This convergence suggests that the additive value of multimodal integration is not tumor-specific but reflects a broader principle in oncologic imaging: that radiomics performs best when anchored by complementary biological and clinical information.

Importantly, the methodological limitations identified in the current literature have direct implications for clinical translation. The predominance of retrospective study designs limits generalizability and constrains the use of radiomics models in prospective clinical decision-making, where real-time risk stratification is required. In addition, the frequent reliance on internal validation alone restricts clinical deployment, as models that have not been externally tested may fail to maintain performance across institutions, scanners, and patient populations. Heterogeneity in radiomics workflows—including variability in image acquisition, segmentation, feature extraction, and model development—further hampers standardization and represents a substantial barrier to regulatory approval and multicenter implementation. Finally, the limited integration of radiomics outputs into established clinical workflows and decision pathways, despite encouraging diagnostic performance, delays adoption and underscores the gap between proof-of-concept studies and routine clinical use.

In practical terms, the most immediate clinical impact of radiomics may lie in the post-chemotherapy assessment of retroperitoneal residual masses, where current imaging lacks specificity and leads to substantial overtreatment. As summarized in the Results, several CT-based radiomics models demonstrated encouraging performance in differentiating necrosis or fibrosis from teratoma or viable tumor, particularly when combined with clinical or molecular markers. In carefully selected patients with favorable radiomics signatures, such models could support more individualized decision-making by identifying those at low likelihood of harboring viable disease, thereby potentially reducing unnecessary retroperitoneal lymph node dissections and their associated morbidity. Importantly, radiomics should be viewed as a complementary decision-support tool rather than a replacement for surgical staging, with its role best suited to refining patient selection in equivocal cases.

Future multicenter radiomics studies in testicular cancer should focus on two key priorities to facilitate clinical translation. First, methodological standardization—including adherence to IBSI-compliant feature definitions and harmonization of imaging acquisition and reconstruction protocols, particularly for CT—is essential to reduce non-biological variability. Second, robust external validation in prospective, multicenter cohorts should be systematically incorporated to ensure generalizability and support integration of radiomics-based tools into routine clinical decision-making.

Taken together, emerging radiomics and multimodal imaging strategies have the potential to transform the diagnostic and therapeutic landscape of testicular cancer. As methodological standards improve and prospective validation expands, these tools may become integral components of precision oncology, enabling more accurate risk stratification and more judicious use of invasive treatments.

## 5. Limitations

This review is subject to certain limitations that merit consideration. First, the available radiomics literature in testicular cancer remains limited by small sample sizes and heterogeneous methodologies, including variations in imaging acquisition, segmentation protocols, feature extraction, and machine-learning approaches, which complicate direct comparison across studies. Second, the evidence base is constrained to published data, introducing the possibility of publication bias and preferential reporting of positive findings. In addition, the review protocol was not prospectively registered, which may increase the risk of selection or reporting bias and limit transparency in study identification and inclusion. Finally, the scarcity of multicenter or prospective validation studies limits the generalizability of current radiomics models. These factors underscore the need for more standardized, collaborative research efforts to clarify the true clinical utility of radiomics in testicular cancer.

## 6. Conclusions

Radiomics and AI-based approaches show meaningful promise for enhancing both primary tumor evaluation and the characterization of post-chemotherapy residual masses in testicular cancer. The most compelling results arise from combined models that integrate quantitative imaging with clinical or molecular data, reflecting the multifaceted biology of these tumors. With appropriate standardization and rigorous prospective validation, such multimodal frameworks have the potential to reduce unnecessary interventions and support more precise, individualized management throughout the disease course.

## Figures and Tables

**Table 1:** Overview of included ultrasound-based radiomics studies in testicular cancer.

No.	Author, Year	Study Design	Sample Size (*n*)	Tumor Type	US Technique	Segmentation	Extracted Features	Ground Truth	Validation	Performance
1.	Fang et al., 2024 [12]	Retrospective Multicenter	275 Training *n* = 158 Internal validation *n* = 68 External validation *n* = 49	Benign versus malignant	Conventional grayscale 4–12 MHz linear probe	Manual	First and second order *n* = 1561	Testicular biopsy Radical orchiectomy	Internal split 70–30% External cohort	Internal validation 85% External validation 80.3%
2.	Lin et al., 2025 [13]	Retrospective Single center	148 Training *n* = 104 Validation *n* = 44	TGCT versus TNGCT SGCT versus NSGCT	Conventional grayscale 4–15 MHz linear probe	Manual	First and second order *n* = 464	Radical orchiectomy	Internal split 70–30%	TGCT versus TNGCT 82% SGCT versus NSGCT 74%
3.	Zhang et al., 2025 [14]	Retrospective Multicenter	489 Training *n* = 338 Internal validation *n* = 92 External validation *n* = 59	SGCT versus NSGCT	Conventional grayscale with super resolution reconstruction 7–15 MHz linear probe	Manual	First and second order *n* = 1552	Radical orchiectomy	Internal 5-fold cross-validation External cohort	Internal validation 91% External validation 82%

US = Ultrasound; TGCT = Testicular germ cell tumor; TNGCT = Testicular non-germ cell tumor; SGCT = Seminomatous germ cell tumor; NSGCT = Non-seminomatous germ cell tumor; MHz = Megahertz.

**Table 2:** Overview of included magnetic resonance imaging-based radiomics studies in testicular cancer.

No.	Author, Year	Study Design	Sample Size (*n*)	Tumor Type	MRI Technique	Segmentation	Extracted Features	Ground Truth	Validation	Performance
1.	Zhang et al., 2019 [15]	Retrospective Single center	39 No separate training and validation cohorts	SGCT versus NSGCT	3 Tesla T2WI used for analysis	Manual	First and second order *n* = 851	Radical orchiectomy	Internal 5-fold cross-validation	SGCT versus NSGCT 97.9%
2.	Feliciani et al. 2021 [16]	Retrospective Single center	44 No separate training and validation cohorts	TGCT versus TNGCT SGCT versus NSGCT	1.5 Tesla T2WI used for analysis	Manual	First, second and high order *n* = 484	Testicular biopsy Radical orchiectomy	Internal 5-fold cross-validation LASSO feature selection SVM models	TGCT versus TNGCT 89% SGCT versus NSGCT 86%
3.	Zhang et al., 2021 [17]	Retrospective Single center	80 No separate training and validation cohorts	Benign versus malignant	3 Tesla T2WI used for analysis	Manual	First order *n* = 66	Radical orchiectomy	Internal validation No dataset split or cross-validation	Benign versus malignant 80.7%
4.	Fan et al., 2022 [18]	Retrospective Single center	101 Training *n* = 71 Validation *n* = 30	Benign versus malignant	3 Tesla ADC used for analysis	Manual	First and second order *n* = 851	Testicular biopsy Radical orchiectomy	Internal split 70–30% Internal 10-fold cross-validation LASSO feature selection	Training 90.4% Validation 86.8%
5.	Feng et al., 2023 [19]	Retrospective Single center	115 Training *n* = 81 Validation *n* = 34	Benign versus malignant	3 Tesla T2WI used for analysis	Manual	First and second order *n* = 1781	Testicular biopsy Radical orchiectomy	Internal split 70–30% Internal 10-fold cross-validation	Training 98.7% Validation 90.5%
6.	Jian et al., 2025 [20]	Retrospective Single center	148 Training *n* = 103 Validation *n* = 45	Benign versus malignant	1.5 Tesla T2WI, ADC, DWI and DCE used for analysis	Manual	First and second order *n* = 1409	Radical orchiectomy	Internal split 70–30% Internal 10-fold cross-validation LASSO feature selection	Benign versus malignant 81%
7.	Wang et al., 2025 [21]	Retrospective Single center	123 Training *n* = 86 Validation *n* = 37	Benign versus malignant	3 Tesla T2WI used for analysis	Manual	First and second order *n* = 1781	Testicular biopsy Radical orchiectomy	Internal split 70–30% Internal 5-fold cross-validation	Training 94.2% Validation 78.4%

T2WI = T2-weighted imaging; ADC = Apparent diffusion coefficient; DWI = Diffusion-weighted imaging; DCE = Dynamic contrast-enhanced; TGCT = Testicular germ cell tumor; TNGCT = Testicular non-germ cell tumor; SGCT = Seminomatous germ cell tumor; NSGCT = Non-seminomatous germ cell tumor; LASSO = Least Absolute Shrinkage and Selection Operator; SVM = Support Vector Machine.

**Table 3:** Overview of included computed tomography-based radiomics studies investigating retroperitoneal lymph nodes and residual mass evaluation.

No.	Author, Year	Study Design	Sample Size (*n*)	Tumor Type	CT Technique	Segmentation	Extracted Features	Ground Truth	Validation	Performance
1.	Lewin et al., 2018 [10]	Retrospective Single center	102 No separate training and validation cohorts	Teratoma versus GCT versus fibrosis/necrosis	Contrast-enhanced CT 5 mm slice thickness	Manual	First and second order *n* = 153	pcRPLND	Nested 10-fold cross-validation, repeated 100 times	Global accuracy 71.7%
2.	Baessler et al., 2020 [22]	Retrospective Multicenter	204 Training *n* = 120 Validation *n* = 23 External validation *n* = 61	Teratoma versus GCT versus fibrosis/necrosis	Contrast-enhanced CT 2–5 mm slice thickness	Semi-automatic	First and second order *n* = 97	pcRPLND	Internal 10-fold cross-validation	Training 96% Validation 81%
3.	Fournier et al., 2023 [23]	Retrospective Single center	149 Training *n* = 99 Validation *n* = 50	Teratoma versus GCT versus fibrosis/necrosis	Contrast-enhanced CT 2 mm slice thickness	Manual	First and second order *n* = 178	Retroperitoneal residual mass resection	Internal split 66–33%	Training 85.6% Validation 82.3%
4.	Lisson et al., 2023 [24]	Retrospective Single center	273 Training *n* = 191 Validation *n* = 82	SGCT versus NSGCT	Contrast-enhanced CT 3 mm slice thickness	Manual	First and second order *n* = 85	Clinical relapse	Internal split 70–30% Internal 10-fold cross-validation SMOTE oversampling for the training datasets	Global accuracy 83%
5.	Scavuzzo et al., 2023 [25]	Retrospective Single center	122 No separate training and validation cohorts	Teratoma versus GCT versus fibrosis/necrosis	Contrast-enhanced CT 2–5 mm slice thickness	Semi-automatic	First and second order *n* = 851	pcRPLND	Internal 5-fold cross-validation, repeated 100 times	Global accuracy 85.3%
6.	Li et al., 2024 [26]	Retrospective (training and internal validation) and prospective (external validation) Multicenter	187 Training *n* = 108 Validation *n* = 37 External validation *n* = 42	Teratoma versus GCT versus fibrosis/necrosis	Contrast-enhanced CT 2–5 mm slice thickness	Manual	First and second order *n* = 1130	pcRPLND	Internal split 70–30%	Global accuracy 81%
7.	Venishetty et al., 2024 [27]	Retrospective Single center	45 No separate training and validation cohorts	Teratoma versus GCT versus fibrosis/necrosis	Contrast-enhanced CT Slice thickness not reported	Manual	First order Total number of features not reported	pcRPLND	Multiple *t*-tests with Benjamini–Hochberg correction	No reported accuracy High collinearity 0.87–0.98
8.	Ozgun et al., 2025 [28]	Retrospective Single center	111 Training *n* = 78 Validation *n* = 33	Teratoma versus non-teratoma	Contrast-enhanced CT Slice thickness not reported	Manual	First and second order Total number of features not reported	pcRPLND	Internal split 70–30% Nested 10-fold cross-validation	Training 89% Validation 81%

GCT = Germ cell tumor; CT = Computed tomography; pcRPLND = Post-chemotherapy retroperitoneal lymph node dissection; SGCT = Seminomatous germ cell tumor; NSGCT = Non-seminomatous germ cell tumor.

**Table 4:** Compact summary of the principal diagnostic tasks addressed by radiomics-based studies in testicular cancer imaging, highlighting the best-reported performance metrics for each imaging modality; detailed methodological characteristics and full performance results are provided in Table 1, Table 2 and Table 3.

Diagnostic Task	Modality	Author, Year	Model	Best Reported Performance	Validation
Benign versus malignant	US	Fang et al., 2024 [12]	Deep learning radiomics model (ResNet-50)	80.3%	External validation
	MRI	Feng et al., 2023 [19]	Machine learning radiomics model (XGBoost)	90.5%	Internal split (train/test) 70–30%
TGCT versus TNGCT	US	Lin et al., 2025 [13]	Machine learning radiomics model (Multivariate Logistic Regression)	82%	Internal split (train/test) 70–30%
	MRI	Feliciani et al. 2021 [16]	Machine learning radiomics model (Support Vector Machine)	89%	Internal 5-fold cross-validation
SGCT versus NSGCT	US	Zhang et al., 2025 [14]	Deep learning (Generative Adversarial Network) radiomics feature extraction and machine learning (Logistic Regression) classifier	82%	External validation
	MRI	Zhang et al., 2019 [15]	Machine learning radiomics model (LASSO Logistic Regression)	97.9%	Internal 5-fold cross-validation
	CT	Lisson et al., 2023 [24]	Machine learning radiomics model (Random Forest)	83%	Internal split (train/test) 70–30% Internal 10-fold cross-validation
Viable retroperitoneal mass versus fibrosis/necrosis	CT	Scavuzzo et al., 2023 [25]	Machine learning radiomics model (Support Vector Machine)	85.3%	Internal 5-fold cross-validation
Retroperitoneal teratoma versus non-teratoma residual disease	CT	Ozgun et al., 2025 [28]	Machine learning radiomics model (CatBoost)	81%	Internal split (train/test) 70–30%

US = Ultrasound; MRI = Magnetic resonance imaging; CT = Computed tomography; TGCT = Testicular germ cell tumor; TNGCT = Testicular non-germ cell tumor; SGCT = Seminomatous germ cell tumor; NSGCT = Non-seminomatous germ cell tumor; LASSO = Least Absolute Shrinkage and Selection Operator.

**Table 5:** Summary of combined radiomic-clinical and radiomic-molecular model characteristics from the previously analyzed studies.

No.	Author, Year	Imaging Modality	Sample Size (*n*)	Tumor Type	Integrated Data Types	Integrate Variables	Prediction Task	Modeling Approach	Validation	Performance
1.	Lisson et al., 2023 [24]	Contrast-enhanced computed tomography	*n* = 273 Retroperitoneal lymph nodes	SGCT and NSGCT	Clinical and biochemical	- Age - AFP - β-hCG - BMI	Preoperative prediction of lymph node metastasis	Random Forest SVM kNN	Internal split 70–30% Internal 10-fold cross-validation SMOTE oversampling	Random Forest combined model 87%
2.	Fang et al., 2024 [12]	Conventional grayscale ultrasound	*n* = 275 Primary testicular lesions	Benign versus malignant	Clinical and biochemical	- Age - AFP - β-hCG - BMI - Scrotal pain - Comorbidities - CBC	Preoperative prediction of benign versus malignant lesions	Deep learning algorithm	Internal split 70–30% External validation cohort	Deep learning combined model 90.9%
3.	Li et al., 2024 [26]	Contrast-enhanced computed tomography	*n* = 187 Retroperitoneal residual masses	Teratoma versus GCT versus fibrosis/necrosis	Serum microRNAs	- miR-371a-3p - miR-375-5p	Pre-operative classification of post-chemotherapy residual masses	Logistic Regression	Internal split 70–30%	Logistic Regression combined model 91%
4.	Jian et al., 2025 [20]	Multiparametric magnetic resonance imaging	*n* = 148 Primary testicular lesions	Benign versus malignant	Clinical and biochemical	- Age - AFP - β-hCG - Size - Laterality	Preoperative prediction of benign versus malignant lesions	Random Forest SVM Logistic Regression kNN	Internal split 70–30% Internal 10-fold cross-validation LASSO feature selection	Logistic Regression combined with tumor size model 88.4%
5.	Lin et al., 2025 [13]	Conventional grayscale ultrasound	*n* = 148 Primary testicular lesions	TGCT versus TNGCT SGCT versus NSGCT	Clinical and biochemical	- Age - AFP - β-hCG - Size - Cryptorchidism history	Preoperative prediction of testicular tumor subtype	Multivariate logistic regression	Internal split 70–30%	Multivariate logistic regression combined model TGCT versus TNGCT 89% SGCT versus NSGCT 86%
6.	Ozgun et al., 2025 [28]	Contrast-enhanced computed tomography	*n* = 111 Retroperitoneal residual masses	Teratoma versus non-teratoma	Clinical, biochemical and serum microRNAs	- Age - AFP - β-hCG - Location - miR-371 - miR-375	Pre-operative classification of post-chemotherapy residual masses	Random Forest SVM CatBoost Gradient Boosting	Internal split 70–30% Nested 10-fold cross-validation	CatBoost combined model 96%

SGCT = Seminomatous germ cell tumor; NSGCT = Non-seminomatous germ cell tumor; TGCT = Testicular germ cell tumor; TNGCT = Testicular non-germ cell tumor; AFP = Alpha-Fetoprotein; β-hCG = Beta-human chorionic gonadotropin; BMI = Body mass index; SVM = Support Vector Machine; kNN = k-nearest neighbor; CBC = Complete blood count.

## Data Availability

The original contributions presented in this study are included in the article. Further inquiries can be directed to the corresponding author.

## Supplementary Materials

The following supporting information can be downloaded at https://www.mdpi.com/article/10.3390/medicina62010125/s1, File S1: PRISMA checklist [39].
